# Fuzzy backstepping controller for agricultural tractor-trailer vehicles path tracking control with experimental validation

**DOI:** 10.3389/fpls.2024.1513544

**Published:** 2024-12-17

**Authors:** Anzhe Wang, Yefei Wang, Xin Ji, Kun Wang, Meiling Qian, Xinhua Wei, Qi Song, Wenming Chen, Shaocen Zhang

**Affiliations:** ^1^ School of Agricultural Engineering, Jiangsu University, Zhenjiang, China; ^2^ College of Mechanical Engineering, Yangzhou University, Yangzhou, China; ^3^ Key Laboratory of Modern Agricultural Equipment and Technology, Ministry of Education, Zhenjiang, China

**Keywords:** agricultural tractor-trailer vehicle, smart planting, path tracking control, back-stepping control, fuzzy control

## Abstract

Unmanned driving technology for agricultural vehicles is pivotal in advancing modern agriculture towards precision, intelligence, and sustainability. Among agricultural machinery, autonomous driving technology for agricultural tractor-trailer vehicles (ATTVs) has garnered significant attention in recent years. ATTVs comprise large implements connected to tractors through hitch points and are extensively utilized in agricultural production. The primary objective of current research focus on autonomous driving technology for tractor-trailers is to enable the tractor to follow a reference path while adhering to constraints imposed by the trailer, which may not always align with agronomic requirements. To address the challenge of path tracking for ATTVs, this paper proposes a fuzzy back-stepping path tracking controller based on the kinematic model of ATTVs. Initially, the path tracking kinematic error model was established with the trailer as the positioning center in the Frenet coordinate system using the velocity decomposition method. Then, the path tracking controller was designed using the back-stepping algorithm to calculate the target front wheel steering angle of the tractor. The gain coefficient was adaptively adjusted through a fuzzy algorithm. Co-simulation and experiments were conducted using MATLAB/Simulink/CarSim and a physical platform, respectively. Simulation results indicated that the proposed controller reduced the trailer's online time by 36.33%. When following a curved path, the trailer's tracking error was significantly lower than that of the Stanley controller designed for a single tractor. In actual experiments, while tracking a U-turn path, the proposed controller reduced the average absolute value of the trailer's path tracking lateral error by 65.27% and the maximum lateral error by 87.54%. The mean absolute error (MAE) values for lateral error and heading error were 0.010 and 0.016, respectively, while the integral of absolute error (IAE) values were 1.989 and 2.916, respectively. The proposed fuzzy back-stepping path tracking controller effectively addresses the practical challenges of ATTV path tracking. By prioritizing the path tracking performance of the trailer, the quality and efficiency of ATTVs during field operations are enhanced. The significant reduction in tracking errors and online time demonstrates the effectiveness of the proposed controller in improving the accuracy and efficiency of ATTVs.

## 1 Introduction

The modern field planting industry is confronted with significant challenges related to labor and land resource shortages, necessitating a transition to smart, precision, and sustainable agricultural practices. (Liu et al., 2019; Thilakarathne et al., 2023; Wang et al., 2023). In this context, intelligent agricultural machinery has been extensively implemented in various agricultural production processes, including sowing and fertilization, leading to substantial increases in crop yields (Xu et al., 2019; Yin et al., 2021; Liang, 2023). Agricultural tractors are the most widely utilized vehicles in field operations and can effectively collaborate with other agricultural machinery to execute a comprehensive range of tasks, from planting to harvesting. Consequently, the automatic driving technology of tractors has been the focus of extensive research in recent years (Sun J. et al., 2023; Ji et al., 2023; Ou et al., 2023). Notably, with the increasing trend towards large-scale agricultural implements, these tools have been adopted across all aspects of agricultural production due to their remarkable attributes, including flexibility and high efficiency. Examples include land levelers, grain transport trailers, seed-fertilizer drill machine, and hydraulic reversible plough (Jing et al., 2020; Zavrazhnov et al., 2023; Nielsen et al., 2017). These implements (henceforth referred to as trailers) and agriculture tractors are interconnected through hitch points to form ATTVs.

**Figure 1:**
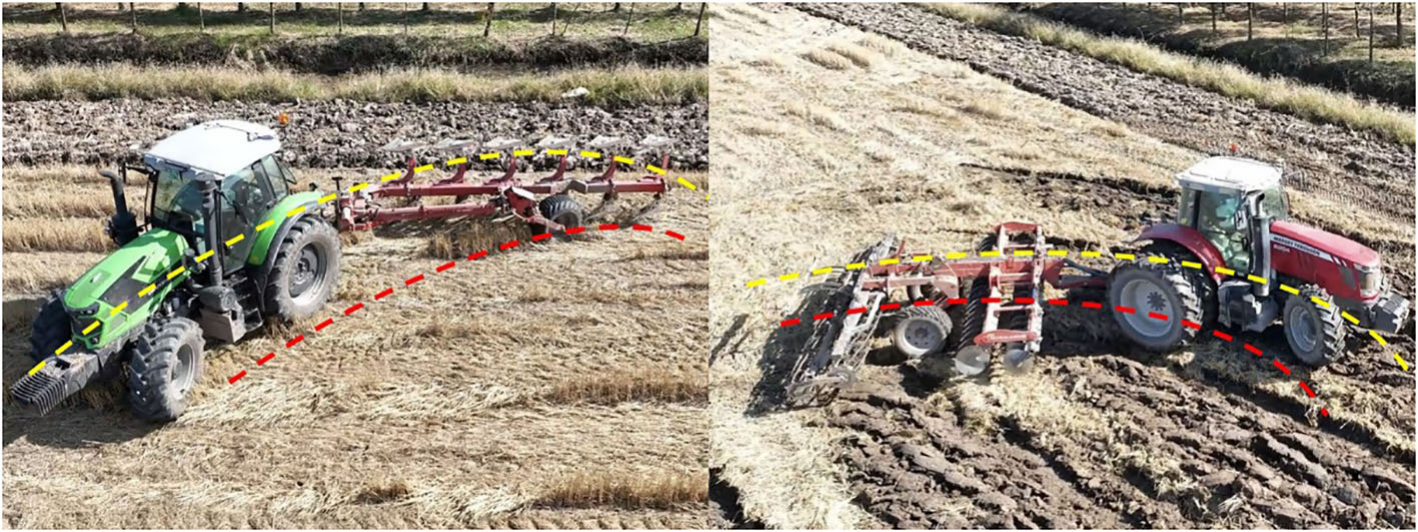
The driving trajectory of tractor and trailer.

As illustrated in **Figure 1**, the yellow line denotes the driving trajectory of the tractor, while the red line indicates the driving trajectory of the trailer. A notable characteristic of the tractor-trailer system is that the trajectories of the tractor and trailer differ during operation. In contrast to tractor-trailer vehicles employed in other industries (Alshaer et al., 2014; Sun N. et al., 2023; Bako, 2021), agricultural trailers must adhere to specific trajectories dictated by agronomic practices, whereas the travel trajectory of the tractor itself is subject to less stringent requirements. Additionally, unlike the path tracking control method employed for a single tractor, the ATTV system can only regulate the tractor’s movement through the front wheel steering angle, which in turn indirectly influences the trailer via the articulation angle. Furthermore, the ATTV system is constrained by physical limitations, including a maximum articulation angle and a maximum front wheel steering angle. These factors pose substantial challenges in the design of an effective path tracking controller.

Numerous scholars have conducted mechanism analyses and modeling of the tractor-trailer system, developed related controllers and achieved significant research results. The study of tractor-trailer systems within the realms of robotics and transport vehicles began early. Many researchers established kinematic models in the Cartesian coordinate system; however, the positioning center of these models predominantly focused on the tractor rather than the trailer (DeSantis, 1994; Binh et al., 2019; Murillo et al., 2022). Furthermore, the primary objective of their research was to enable the tractor to follow a reference path, treating the trailer, which is towed behind the tractor, merely as an external constraint to fulfill this goal. This approach does not align with the specific requirements of the agricultural sector. To enhance trailer control, some researchers have installed electro-hydraulic valve actuators at the hitch points. These actuators work in conjunction with inductive sensors to create a lower-level controller that adjusts the tractor based on the target articulation angle provided by an upper-level controller, thus ensuring the necessary stance for the trailer combination (Kayacan et al., 2014; Bai et al., 2019). It is undeniable that adding a steering actuator at the hitch point can significantly enhance the movement accuracy and maneuverability of the unit. However, for ATTV, the implementation challenges and associated costs of this technology are relatively high, particularly in bumpy farmland scenarios. Establishing an actuator-less vehicle model offers a cost-effective alternative for ATTV path tracking control. Some researchers conceptualize the tractor within the ATTV system as a two-wheeled robot model. By employing intelligent algorithms such as Model Predictive Control (MPC), Linear Quadratic Regulator (LQR), and neural networks, the target speeds for the left and right wheels can be calculated (Yue et al., 2018; Shojaei, 2021; Lu et al., 2023). The speed differential between the side wheels generates torque, which subsequently drives the trailer for path tracking control. However, in practical ATTV usage scenarios, we can only control the steering angle of the tractor’s front wheel, while the speed difference between the left and right wheels remains uncontrollable. Consequently, these control methods cannot yet be implemented in real systems. Learning or evolution-based methods are employed in certain autonomous driving algorithms (Hougen et al., 1997; Bachute and Subhedar, 2021); however, these approaches typically require substantial amounts of time and data to train the controller when applied to actual vehicles. Furthermore, they are not suitable for ATTV autonomous driving involving various combinations of tractors and trailers. The kinematic monorail model features a simple structure and high accuracy at low speeds, making it suitable for agricultural machinery operations (Huynh et al., 2010; Ji et al., 2024). Some researchers have developed ATTV models based on this framework and designed controllers accordingly. In (Astolfi et al., 2004), the kinematic models of tractors and trailers were established in the Cartesian coordinate system. In (Huang et al., 2023), based on the existing kinematic model and further integrating the desired linear path expression, a linear path tracking controller for ATTV was developed using the sliding mode algorithm.

In summary, the control objectives of this paper can be articulated as follows:

- When the ATTV follows a straight reference path, our objective is to ensure that the trailer tracks the reference path (both the lateral and heading errors converge to zero) before the tractor does, rather than allowing the trailer’s tracking error to gradually converge only after the tractor comes online.
- When the ATTV follows a curved reference path—where the trajectories of the tractor and trailer do not overlap—our objective is to ensure that the trailer tracks the reference path.

To achieve the aforementioned objectives, this paper presents a novel controller developed using the back-stepping algorithm and fuzzy logic to address the trailer path tracking problem in ATTV systems. The primary contributions and innovations of this paper are (1) The kinematic error model of the ATTV was established using the velocity decomposition method within Frenet coordinates, leading to the derivation of the system state equation with the trailer as the positioning center. This model significantly enhances the design of a subsequent tracking controller. Furthermore, the constraints associated with the relevant physical parameters in the model are presented. Notably, in contrast to many prior studies (Yue et al., 2018; Shojaei, 2021; Lu et al., 2023), the control variable of this model is the front wheel steering angle of the tractor, thereby ensuring that the algorithm is applicable to real-world vehicles; (2) A path tracking controller for the trailer is developed based on backstepping theory, with stability demonstrated through the Lyapunov method. This controller guarantees that the trailer adheres to the reference path, resulting in the lateral, heading, and articulation angle errors asymptotically converging to zero; (3) The fuzzy algorithm is integrated with the back-stepping controller to adaptively adjust the gain coefficient of the controller, thereby enhancing convergence speed and minimizing overshoot errors.

## 2 Materials and methods

### 2.1 System description and modeling

**Figure 2:**
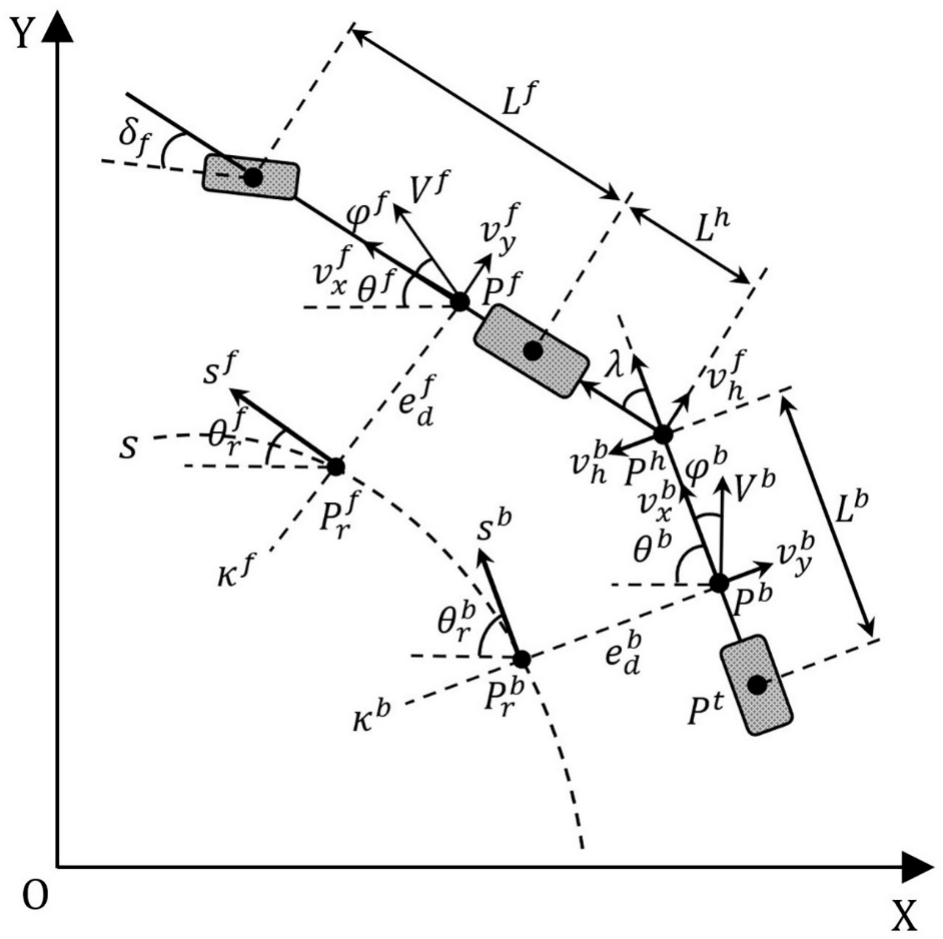
The kinematic single-track model of the ATTV.

As illustrated in **Figure 2**, the kinematic single-track model of the ATTV is developed employing the velocity decomposition method. The XOY coordinate system denotes the inertial coordinate frame, while the reference trajectory is formulated within the Frenet framework. The three wheels, from left to right, comprise the front wheel of the tractor, the rear wheel of the tractor, and the trailer wheel. The tractor and trailer are connected at the hinge point $P^{h}$ . The tractor adjusts the front wheel steering angle $\delta_{f}$ to ensure that the trailer’s center of mass point $P^{b}$ follows the reference path s . Furthermore, **Table 1** presents a detailed list of the essential parameters, variables, and notations relevant to the model.

**Table 1:** The parameters and variables in the kinematic model.

Parameters and variables	Description	Unit
$P^{f}$	The center of mass of the tractor	/
$P^{h}$	Hitch point	/
$P^{b}$	The center of mass of the trailer	/
$P^{t}$	The rear axle center of the trailer	/
s	The reference path	/
$P_{r}^{f}$	The projection point of the tractor’s center of mass on the reference path	/
$P_{r}^{b}$	The projection point of the trailer’s center of mass on the reference path	/
$s^{f}$	Tangent vector of curve s at projection point $P_{r}^{f}$	/
$s^{b}$	Tangent vector of curve s at projection point $P_{r}^{b}$	/
$L^{f}$	The wheelbase of the tractor	m
$L^{b}$	The wheelbase of the trailer	m
$L^{h}$	The distance from the hitch point to the rear axle of the tractor	m
$e_{d}^{f}$	The lateral error of the tractor	m
$e_{d}^{b}$	The lateral error of the trailer	m
$V^{f}$	The velocity of the tractor	m/s
$V^{b}$	The velocity of the trailer	m/s
$v_{x}^{f}$	The longitudinal velocity component of the tractor	m/s
$v_{y}^{f}$	The lateral velocity component of the tractor	m/s
$v_{x}^{b}$	The longitudinal velocity component of the trailer	m/s
$v_{y}^{b}$	The lateral velocity component of the trailer	m/s
$v_{h}^{f}$	The lateral velocity component of the hitch point along the tractor	m/s
$v_{h}^{b}$	The lateral velocity component of the hitch point along the trailer	m/s
$e_{\theta }^{f}$	The heading error of the tractor	rad
$e_{\theta }^{b}$	The heading error of the trailer	rad
$\varphi^{f}$	The sideslip angle of the tractor’s center of mass	rad
$\varphi^{b}$	The sideslip angle of the trailer’s center of mass	rad
$\theta^{f}$	The heading angle of the tractor	rad
$\theta^{b}$	The heading angle of the trailer	rad
$\delta_{f}$	The front wheel steering angle of the tractor	rad
λ	The articulation angle	rad
$\kappa^{f}$	Path curvature at projection point $P_{r}^{f}$	1/m
$\kappa^{b}$	Path curvature at projection point $P_{r}^{b}$	1/m

#### 2.1.1 Kinematic error model of ATTV

Assuming that the forward motion of the ATTV occurs on a two-dimensional plane XOY, while neglecting pitch and roll motions, and considering that the left and right tires move symmetrically, a path tracking error model can be established within the Frenet frame:


(1) $$\left\{ \begin{array}{l} {\dot{e}}_{d}^{f}=\left|V^{f}\right|\text{sin }\left(\varphi^{f}+\theta^{f}-\theta_{r}^{f}\right)\\ {\dot{e}}_{\theta }^{f}={\dot{\theta }}^{f}-\left|V^{f}\right|\kappa^{f}\text{cos }\left(\varphi^{f}+\theta^{f}-\theta_{r}^{f}\right)/\left(1-\kappa^{f}e_{d}^{f}\right)\\ {\dot{e}}_{d}^{b}=\left|V^{b}\right|\text{sin }\left(\varphi^{b}+\theta^{b}-\theta_{r}^{b}\right)\\ {\dot{e}}_{\theta }^{b}={\dot{\theta }}^{b}-\left|V^{b}\right|\kappa^{b}\text{cos}\left(\varphi^{b}+\theta^{b}-\theta_{r}^{b}\right)/\left(1-\kappa^{b}e_{d}^{b}\right) \end{array}\right.$$

The error model can be described as follows:


(2) $$\left\{ \begin{array}{l} {\dot{e}}_{d}^{f}=v_{y}^{f}\text{cos}e_{\theta }^{f}+v_{x}^{f}\text{sin}e_{\theta }^{f}\\ {\dot{e}}_{\theta }^{f}={\dot{\theta }}^{f}-\kappa^{f}\left(v_{x}^{f}\text{cos}e_{\theta }^{f}-v_{y}^{f}\text{sin}e_{\theta }^{f}\right)/\left(1-\kappa^{f}e_{d}^{f}\right)\\ {\dot{e}}_{d}^{b}=v_{y}^{b}\text{cos}e_{\theta }^{b}+v_{x}^{b}\text{sin}e_{\theta }^{b}\\ {\dot{e}}_{\theta }^{b}={\dot{\theta }}^{b}-\kappa^{b}\left(v_{x}^{b}\text{cos}e_{\theta }^{b}-v_{y}^{b}\text{sin}e_{\theta }^{b}\right)/\left(1-\kappa^{b}e_{d}^{b}\right) \end{array}\right.$$

When the tractor turns, the trailer is propelled by the traction force at the hitch point $P^{h}$ , rotating around the point $P^{t}$ . The steering speed is determined by the component of the hitch point’s velocity in the lateral direction of the tractor, denoted as $v_{h}^{b}$ . The angular velocity of the heading angle can be expressed as follows:


(3) $${\dot{\theta }}^{b}=\frac{v_{h}^{f}}{L^{b}}=\frac{v_{h}^{f}\text{cos}\lambda -v_{x}^{f}\text{sin}\lambda }{L^{b}}$$

As the trailer follows the reference path, both the lateral and heading errors, along with their derivatives, tend to zero. Simultaneously, the articulation angle converges to the intended target articulation angle. The target articulation angle can be easily determined as:


(4) $$\text{tan}\lambda_{r}=-\kappa^{b}L^{b}$$

In contrast to the trailer, when the tracking error of the tractor is zero, the target front wheel steering angle $\delta_{f,r}$ and the articulation angle $\lambda_{r}^{f}$ are as follows:


(5) $$\text{sin}\delta_{f,r}\;=-\kappa^{f}L^{f}$$

(6) $$\text{tan}\lambda_{r}^{f}=-\kappa^{f}L^{b}$$

Clearly, $\lambda_{r}$ and $\lambda_{r}^{f}$ are not equal when κ≠0 (indicating that the reference path is not a straight line). Therefore, it is not feasible for both the tractor and the trailer to simultaneously track the same reference path. Given that our control objective is to ensure the trailer follows the reference path, we separately extract the last two terms from Equation 2, and substitute Equation 3 into it. Furthermore, during actual field operations, the speed of the ATTV is relatively low, allowing us to neglect the lateral velocity component.


(7) $$\{\begin{array}{c} {\dot{e}}_{d}^{b}=v_{x}^{f}\mathrm{cos\lambda}\text{sin}e_{\theta }^{b}\\ {\dot{e}}_{\theta }^{b}=-\frac{v_{x}^{f}\text{sin}\lambda }{L^{b}}-\frac{\kappa^{b}v_{x}^{f}\text{cosλcos}e_{\theta }^{b}}{1-\kappa^{b}e_{d}^{b}} \end{array}$$

Define the virtual articulation angle error $e_{\lambda }^{b}$ as:


(8) $$\text{tan}e_{\lambda }^{b}=\text{tan}\lambda -\text{tan}\lambda_{r}$$

Therefore, the tracking error model of the trailer can be reformulated as follows:


(9) $$\{\begin{array}{c} {\dot{e}}_{d}^{b}=v_{x}^{b}\text{sin}e_{\theta }^{b}\\ {\dot{e}}_{\theta }^{b}=v_{x}^{b}\kappa^{b}\left(1-\frac{\text{cos}e_{\theta }^{b}}{1-\kappa^{b}e_{d}^{b}}\right)-\frac{v_{x}^{b}\text{tan}e_{\lambda }^{b}}{L^{b}}\\ {\dot{e}}_{\lambda }^{b}=v_{x}^{b}\left(\kappa^{b}-\frac{\text{tan}e_{\lambda }^{b}}{L^{b}}\right)-\frac{v_{x}^{f}\text{tan}\delta_{f}}{L^{f}} \end{array}$$

#### 2.1.2 Physical constraints of parameters

When the steering angle of the tractor’s front wheels is excessively large, the rear profile of the tractor may collide with the front profile of the trailer, resulting in a ‘jack-knife’ phenomenon (Beglini et al., 2020; Zhao et al., 2020). To mitigate this issue, it is essential to impose constraints on both the curvature of the reference path and the steering angle of the tractor’s front wheel prior to operation.

**Figure 3:**
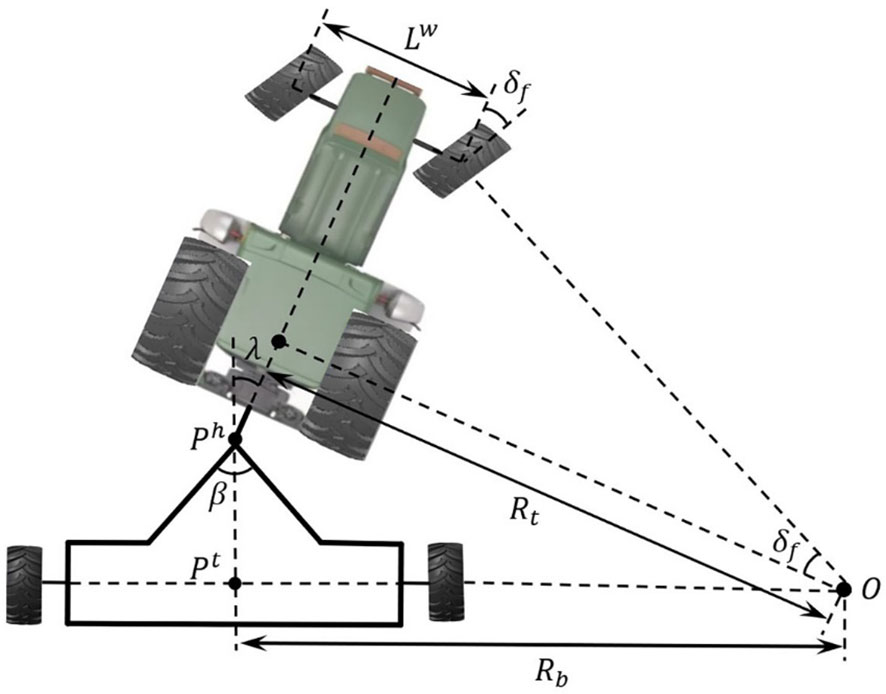
Sketch of the physical constraints of ATTVs.


As illustrated in **Figure 3**, the instantaneous center of rotation for the trailer is denoted as O, while the instantaneous turning radius is represented by $R_{b}$ . At the hitch point $P^{h}$ , the trailer exhibits a physical outline angle, β . To prevent the occurrence of the “jack-knife” phenomenon, the turning radius of the planned reference path R and the front wheel steering angle of the tractor $\delta_{f}$ must adhere to the following constraints prior to path tracking:

(10) $$s.t.\{\begin{array}{c} \left|R\right|>max\left\{\left|R_{min}^{b}\right|,\left|R_{min}^{f}\right|\right\}\\ \left|\delta_{f}\right|<min\left\{\left|\;\delta_{max}^{b}\right|,\left|\;\delta_{max}^{f}\right|\right\} \end{array}$$

Where $R_{min}^{b}$ and $\delta_{max}^{b}$ represent the minimum turning radius of the planned path and the maximum front wheel turning angle of the tractor, respectively, as derived from geometric relationships. $R_{min}^{f}$ and $\delta_{max}^{f}$ denote the minimum turning radius and maximum front wheel steering angle, which are determined by the tractor’s intrinsic mechanical structures at the time of manufacture. The specific mathematical demonstrations can be found in Appendix A.

### 2.2 Path tracking controller design

**Figure 4:**
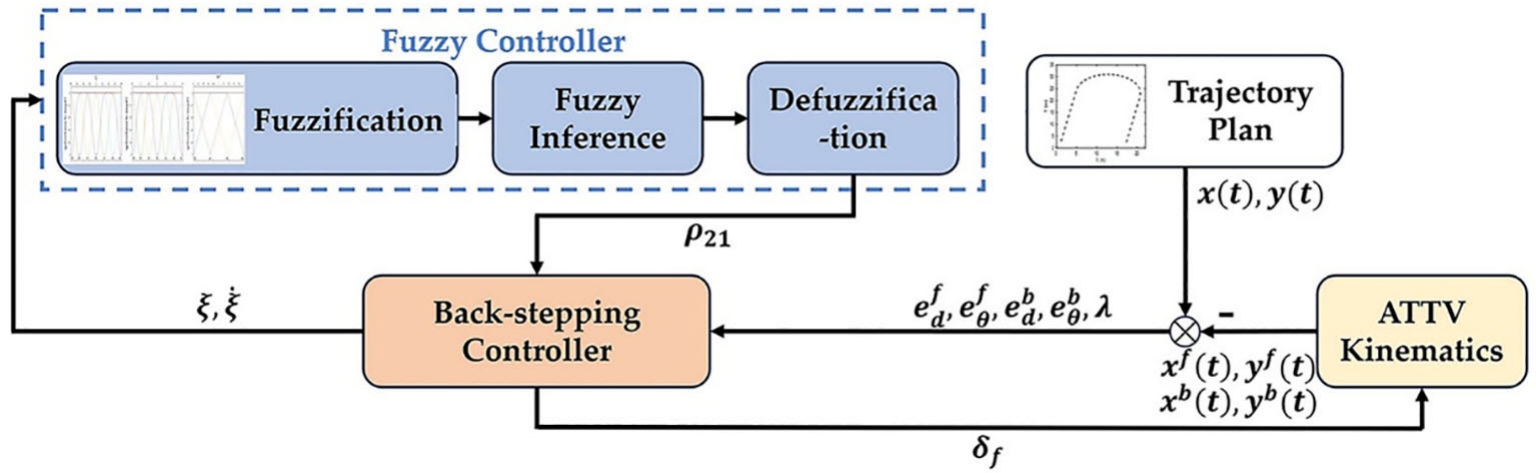
The control frame of fuzzy back-stepping controller.

In this section, we will design a controller for trailer path tracking, with the objective of ensuring that the tracking error of the trailer converges to zero before that of the tractor. The control structure is illustrated in **Figure 4**. Initially, the current position coordinates of the tractor and trailer are obtained based on the kinematics of the ATTV. By comparing these coordinates with the planned reference trajectory, we can ascertain the lateral and heading errors, as well as the articulation angle of the tractor and trailer at their current positions. These error metrics are subsequently transmitted as inputs to the back-stepping controller. The back-stepping controller then calculates the front wheel steering angle of the tractor based on these errors. Concurrently, it sends the articulation angle error and its rate of change to the fuzzy controller, which adaptively tunes the parameters to be fed back to the back-stepping controller.

#### 2.2.1 Back-stepping path tracking controller

To facilitate controller design, the state variable x is defined as $x={\left[x_{1},x_{2},x_{3}\right]}^{T}=\left[e_{d}^{b},e_{\theta }^{b},e_{\lambda }^{b}\right]$ . Subsequently, Equation 9 can be represented in the state-space form as follows:


(11) $$\{\begin{array}{c} {\dot{x}}_{1}=v_{x}^{b}\text{sin}x_{2}\\ {\dot{x}}_{2}=v_{x}^{b}\kappa^{b}\left(1-\frac{\text{cos}x_{2}}{1-\kappa^{b}x_{1}}\right)-\frac{v_{x}^{b}\text{tan}x_{3}}{L^{b}}\\ {\dot{x}}_{3}=v_{x}^{b}\left(\kappa^{b}-\frac{\text{tan}x_{3}}{L^{b}}\right)-\frac{v_{x}^{f}u}{L^{f}} \end{array}$$

where $u=\text{tan}\delta_{f}$ is the system control input.

By examining the system state equation, the state variable $x_{3}$ can be considered as a virtual input in subsystem ${\left[x_{1},x_{2}\right]}^{T}$ . Below, a backstepping method is proposed to attain uniform asymptotic stability in the cascaded system.

**Step 1**: For subsystem ${\left[x_{1},x_{2}\right]}^{T}$ , define a Lyapunov function as:


(12) $$V_{1}=\frac{\rho_{1}\text{ln}\left(\text{cosh}x_{1}\right)}{L^{b}}+\frac{{x_{2}}^{2}}{2}$$

where $\rho_{1}>0$ is the adjustable gain coefficient.

The derivation of $V_{1}$ is:


(13) $${\dot{V}}_{1}=\frac{\rho_{1}v_{x}^{b}\text{tanh}x_{1}\text{sin}x_{2}}{L^{b}}+v_{x}^{b}x_{2}\left\{\kappa^{b}\left(1-\frac{\text{cos}x_{2}}{1-\kappa^{b}x_{1}}\right)-\frac{\text{tan}x_{3}}{L^{b}}\right\}$$

To ensure that ${\dot{V}}_{1}$ is negative semidefinite, we make


(14) $$x_{3,r}=\tan^{-1}\left\{\frac{\rho_{1}\tanh x_{1}\sin x_{2}}{x_{2}}+\tanh x_{2}+L^{b}\kappa^{b}\left(1-\frac{\text{cos}x_{2}}{1-\kappa^{b}x_{1}}\right)\right\}$$

**Step 2**: Let the tracking error ξ as:


(15) $$\xi =x_{3,r}-x_{3}$$

Define a new Lyapunov function as:


(16) $$V_{2}=\frac{\xi^{2}}{2}$$

The derivation of $V_{2}$ is:


(17) $${\dot{V}}_{2}=\xi \left({\dot{x}}_{3,r}+\frac{v_{x}^{b}u}{L^{f}\cos\lambda }+\frac{v_{x}^{b}\tan x_{3}}{L^{b}}-v_{x}^{b}\kappa^{b}\right)$$

To ensure that ${\dot{V}}_{2}$ is negative definite, we make


(18) $$u=\frac{L^{f}\cos\lambda }{v_{x}^{b}}\left(v_{x}^{b}\kappa^{b}-\rho_{2}\xi -{\dot{x}}_{3,r}-\frac{v_{x}^{b}\tan x_{3}}{L^{b}}\right)$$

where $\rho_{2}>0$ is the adjustable gain coefficient.

The mathematical demonstrations regarding the stability proof of the controller can be found in Appendix B.

#### 2.2.2 Adaptive parameter tuning based on fuzzy algorithm

According to Equation 18, the gain coefficient dictates the extent to which articulation angle error affects the front wheel steering angle. A larger gain coefficient can quickly diminish lateral error, enabling the trailer to closely adhere to the reference path. However, if the gain coefficient is excessively large, it may induce oscillations during straight line tracking. In contrast, a smaller gain coefficient facilitates smoother trailer operation and minimizes the steady-state tracking error once the algorithm converges, but the system’s convergence speed may be compromised when faced with a significant initial error. Therefore, the selection of an appropriate gain coefficient should be based on both the magnitude and the rate of change of the articulation angle error. To streamline the parameter tuning process, Equation 18 is further refined as follows:


(19) $$u=\frac{L^{f}\cos\lambda }{v_{x}^{b}}\left(v_{x}^{b}\kappa^{b}-\rho_{21}\rho_{20}\xi -{\dot{x}}_{3,r}-\frac{v_{x}^{b}\tan x_{3}}{L^{b}}\right)$$

where $\rho_{21}$ is the output value of the fuzzy controller, and $\rho_{20}$ represents the manually set initial value of the gain coefficient.

By analyzing the state of ATTV during its driving process, fuzzy rules as presented in **Table 2** are formulated. In these rules, the tracking error ξ and its rate of change are utilized as input variables, while the control parameter $\rho_{21}$ is the output variable. The terms NB, NM, NS, ZO, PS, PM, and PB are defined to represent negative big, negative medium, negative small, zero, positive small, positive medium, and positive big, respectively.

**Table 2:** The fuzzy control rules.

$\dot{\xi }$	ξ						
	NB	NM	NS	ZO	PS	PM	PB
NB	PB	PM	PM	PS	PM	PM	PB
NM	PM	PM	PS	ZO	PS	PM	PM
NS	PM	PS	PS	ZO	PS	PS	PM
ZO	PB	PM	PS	ZO	PS	MS	PB
PS	PM	PS	PS	ZO	PS	PS	PM
PM	PM	PM	PS	ZO	PS	PM	PM
PB	PB	PM	PM	PS	PM	PM	PB

**Figure 5:**
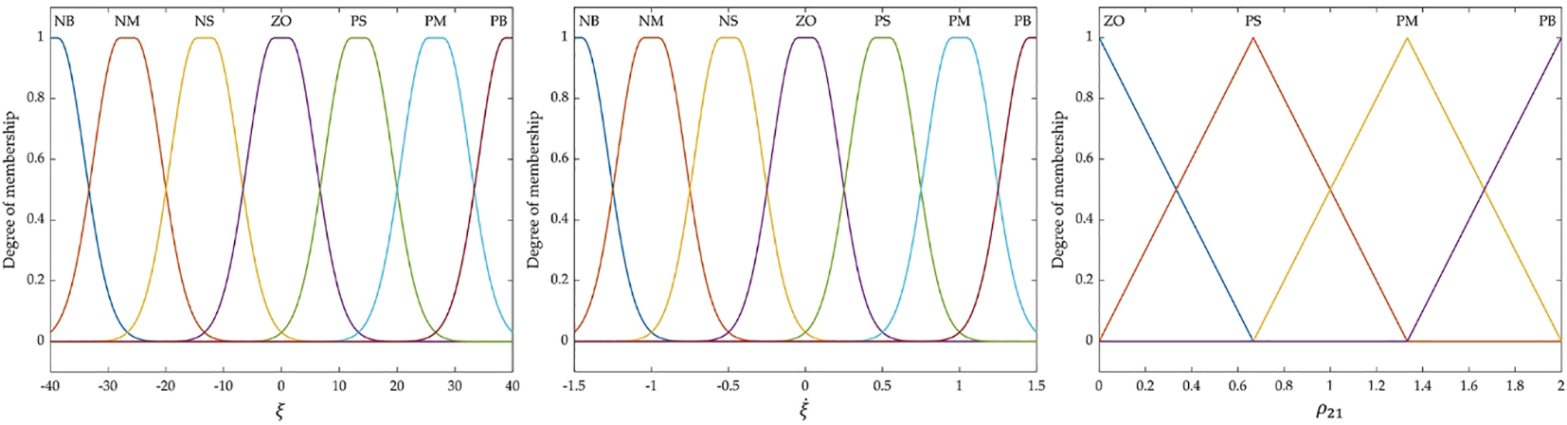
Membership functions of ξ , $\dot{\xi }$ and $\rho_{21}$ .

The fuzzy domain of ξ is set to [−40°,  40°] , which includes seven fuzzy subsets. Similarly, the fuzzy domain of $\dot{\xi }$ is set to [−1.5, 1.5] , also comprising seven fuzzy subsets. The fuzzy domain of the gain coefficient $\rho_{21}$ is set within the range of [0, 2] , consisting of five fuzzy subsets. Their membership functions are illustrated in **Figure 5**. Lastly, the center of gravity method has been selected for the process of defuzzification.

## 3 Results

### 3.1 Co-simulation results

#### 3.1.1 Simulation environment

In order to verify the effectiveness of the proposed controller, we conduct a co-simulation using Matlab Simulink and CarSim. The physical parameters of the ATTV are configured in CarSim as: $L^{f}=3.8$ , $L^{b}=2$ , $L^{h}=0.45$ , and $L^{w}=2.1$ . These parameters are consistent with the specifications of 110 horsepower tractors that are commonly utilized on farms, as well as certain articulated agricultural implements.

In CarSim, a tractor model is designated as the leading vehicle, while a towed single-axle trailer functions as the trailing vehicle. These two vehicles are linked through a rear off-axis single-point articulation, which aligns with the “SA_SA+S” mathematical model form. The tractor’s steering operates under open-loop control, with steering commands computed by the Simulink platform and transmitted with a 0.5 s delay to replicate the time lag characteristic of actual steering mechanisms. The tractor’s speed is maintained at 1 m/s, and the co-simulation step size is set to 0.001 s. To more accurately simulate a real navigation and positioning system, the reference path is discretized into coordinate points spaced 0.1 meters apart. In Simulink, by indexing the navigation point closest to the vehicle, errors are computed between this point and the current vehicle pose state, resulting in the error state variables necessary for the controller. Various error data are gathered at intervals of 0.5 s.

To demonstrate the advantages of the fuzzy back-stepping controller developed for trailers in this paper, we select two controllers as control groups: (1) The back-stepping controller designed for trailer path tracking, with the control input specified by Equation 18. (2) The Stanley controller designed for tractor path tracking, which control input is defined as follows:


(20) $$\delta_{f}=\theta^{f}+\tan^{-1}\frac{\rho_{3}e_{d}^{f}}{V^{f}}$$

In order to facilitate a just comparison during the experiments, the parameters of the control schemes in subsequent work were carefully adjusted several times to reach their best performance.

To verify the effectiveness of the algorithm in achieving the control objectives outlined in the introduction chapter, we selected a long straight line and an arc with a radius of 15 m as reference paths for conducting path tracking simulation experiments. Additionally, to assess the convergence of the tracking error, we ensured that there was a distance between the initial position of the ATTV and the reference path.

#### 3.1.2 Straight path simulation

The reference path is defined as a 30 m straight line along the y=0 axis. The initial position of the tractor is set at (0, -1 m), with initial lateral error, heading error, and articulation angle all set to zero. The coefficients of the three controllers are set to: $\rho_{1}=4.6$ , $\rho_{2}=\rho_{20}=2.5$ , and $\rho_{3}=1.8$ .

**Figure 6:**
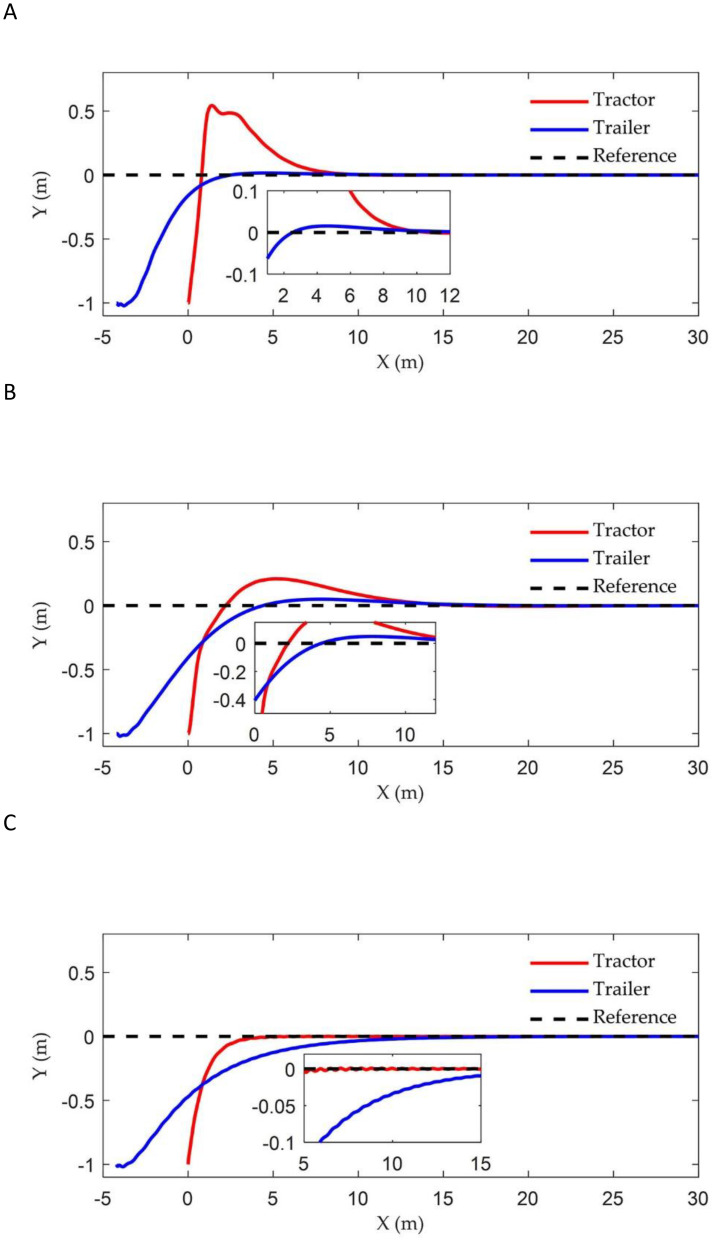
Straight path tracking trajectory of three controllers: **(A)** Fuzzy back-stepping controller; **(B)** Back-stepping controller; **(C)** Stanley controller.

**Figure 7:**
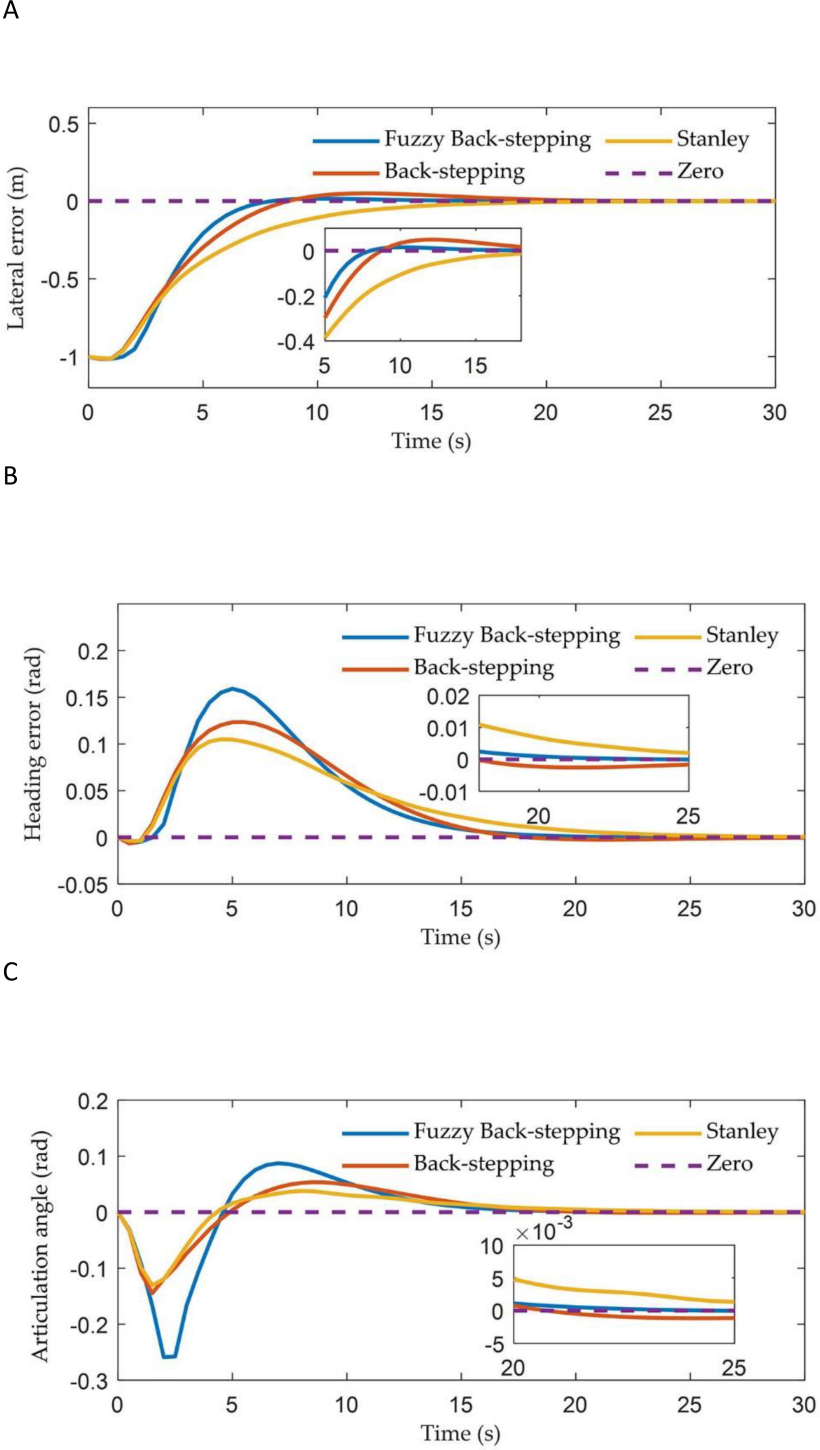
The vehicle states of three controllers in straight path simulation: **(A)** The lateral error; **(B)** The heading error; **(C)** The articulation angle.

**Figure 6** illustrates the tracking trajectories of ATTV under the control of three different controllers. **Figure 7** depicts the history of the lateral error, heading angle error, and articulation angle for these controllers. To quantitatively assess the path tracking capabilities of the three controller, performance indices are defined as the mean absolute error (MAE) $\frac{1}{N}\sum_{k=1}^{N}\left|e\left(k\right)\right|$ and the integral absolute error (IAE) $\sum_{k=1}^{N}\left(\left|e\left(k\right)\right|\text{Δ}t\right)$ . The MAE of the lateral errors for the three types of controllers during the path tracking process is 0.104, 0.115, and 0.135, respectively, while the IAE are 4.201, 4.666, and 5.463, respectively. Similarly, for the heading errors during the path tracking process, the MAE for the three types of controllers are 0.023, 0.025, and 0.026, respectively, with the corresponding IAE being 0.984, 1.024, and 1.051, respectively.

#### 3.1.3 Curve path simulation

**Figure 8:**
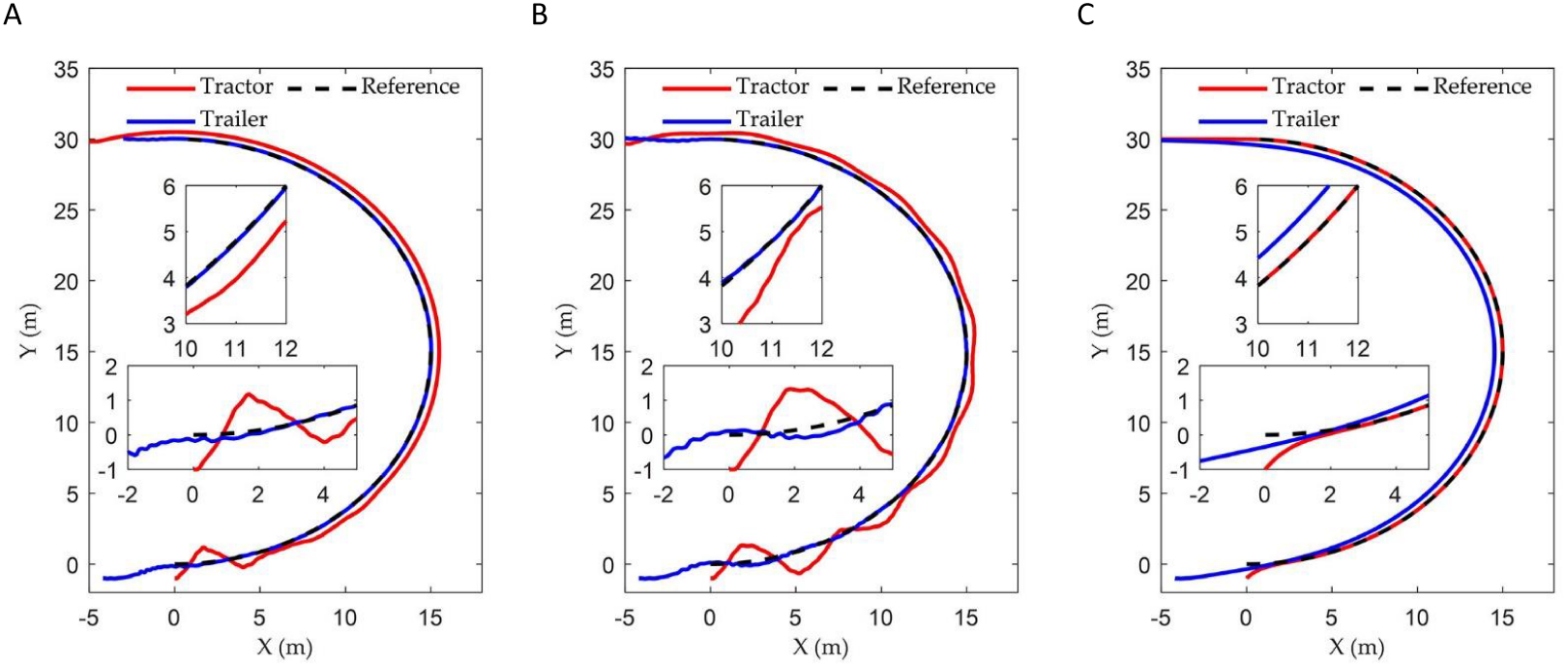
Curve path tracking trajectory of three controllers: **(A)** Fuzzy back-stepping controller; **(B)** Back-stepping controller; **(C)** Stanley controller.

**Figure 9:**
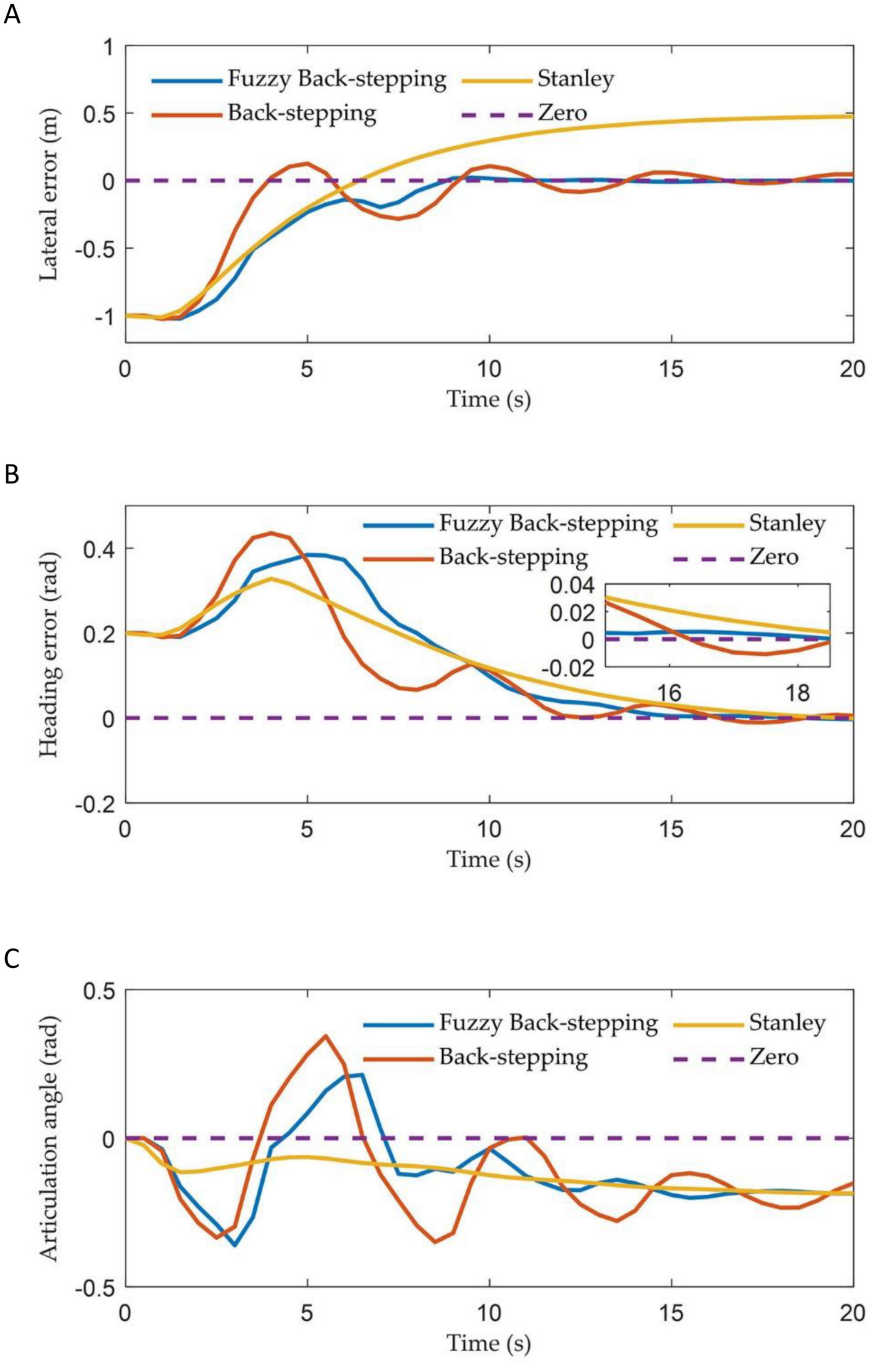
The vehicle states of three controllers in curve path simulation: **(A)** The lateral error; **(B)** The heading error; **(C)** The articulation angle.

The reference path is defined as a semicircular curve with its centre located at the point (0, 15 m) and a radius of 15 m. The initial position of the tractor is established at (0, -1 m), with the initial lateral error, heading error, and articulation angle all set to zero. The coefficients of the three controllers are set to: $\rho_{1}=5$ , $\rho_{2}=\rho_{20}=3.2$ , and $\rho_{3}=2.5$ . **Figure 8** illustrates the tracking trajectories of ATTV under the control of three distinct controllers. **Figure 9** depicts the history of the lateral error, heading angle error, and articulation angle for these three controllers. The MAE of the lateral errors for the three types of controllers during the path tracking process is 0.090, 0.095, and 0.406, respectively, while the IAE are 5.469, 5.767, and 24.536, respectively. Similarly, for the heading errors during the path tracking process, the MAE values for the three types of controllers are 0.158, 0.164, and 0.176, respectively, with the corresponding IAE being 9.582, 9.974, and 10.636, respectively.

### 3.2 Experimental results

**Figure 10:**
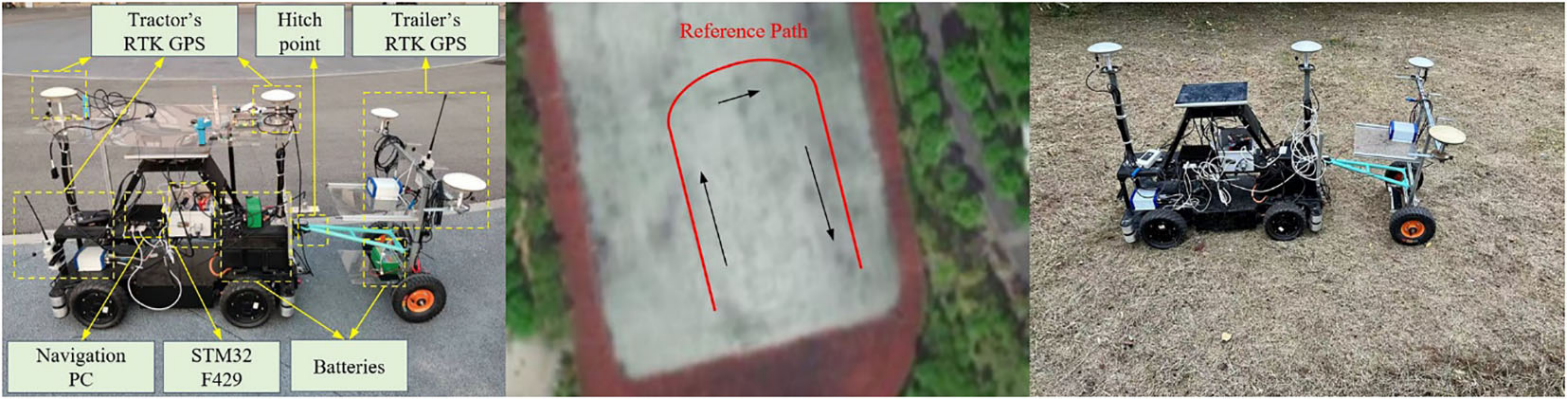
The experimental platform, reference path and environment utilized in the experiment.

This section focuses on experiments conducted on an ATTV-like platform, which comprises a rear-wheel-drive, front-wheel-steering electric car and an unpowered trailer connected via a hitch point. It is important to note that the experimental setup is very representative since it possesses an identical physical framework and electrical design as the large-scale ATTVs widely utilized in agricultural engineering. The experimental platform, resembling ATTVs, mainly comprises RTK-GPS system, radio communication, STM32 microcontroller, and a navigation computer. This arrangement corresponds with the prevailing trends in unmanned agricultural vehicles. Platform photos and planned paths are shown in **Figure 10**. The experiment was conducted in a grassland area covered with soil, closely resembling an actual farmland environment. The platform operates at a constant speed of 1 meter per second, powered by drive motors. The steering angle of the tractor’s front wheel can be adjusted within a range of -35 to 35 degrees. The navigation controller integrates a host PC with two sets of BeiDou RTK satellite navigation systems, which provide lateral and heading errors for both the tractor and the trailer at a frequency of 10 Hz. The articulation angle is calculated from the difference between the heading errors. The STM32 F429 microcontroller functions as the path tracking controller. The reference path comprises three straight line segments and two circular arcs. The coefficients of the three controllers are set to: $\rho_{1}=4.8$ , $\rho_{2}=\rho_{20}=2.1$ , and $\rho_{3}=2$ . The reference path is set to the classic U-turn path commonly utilized in agricultural engineering practices (He et al., 2023). The headland U-turning pattern enables the ATTV to transition to the subsequent row of crops, thereby facilitating ongoing farming operations.

**Figure 11:**
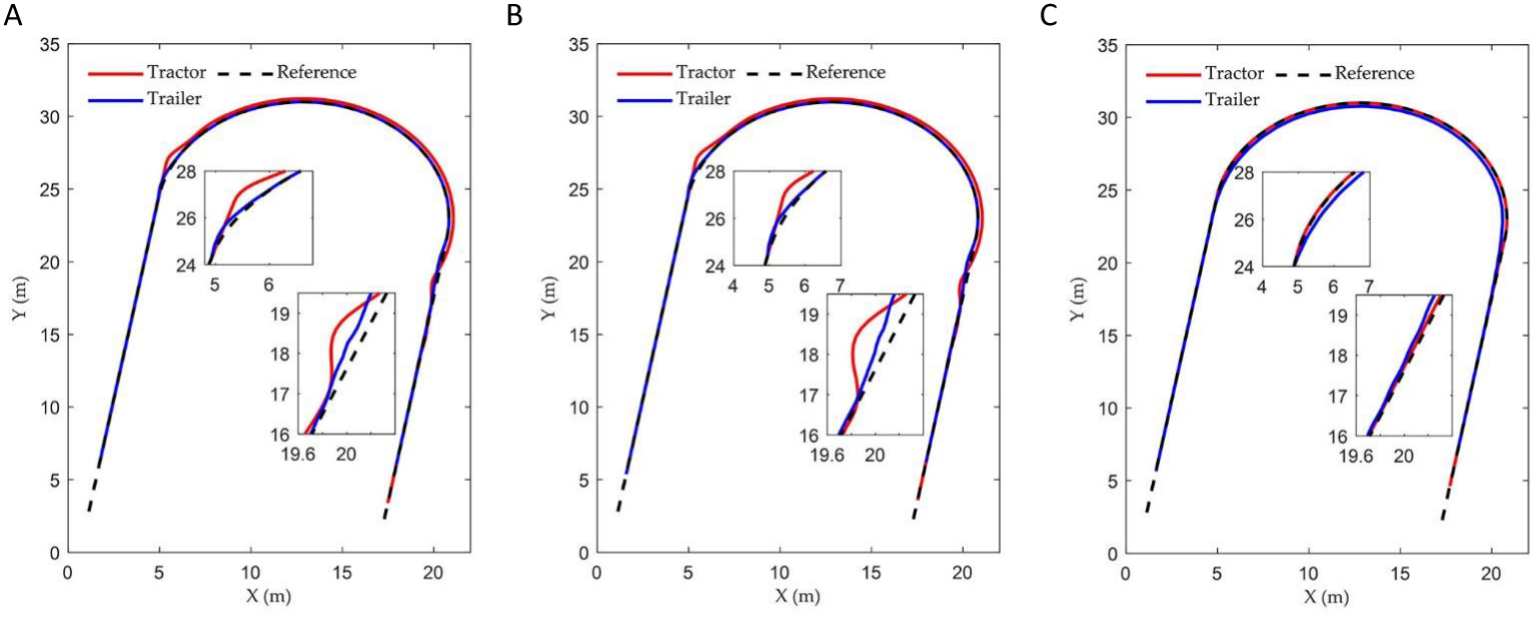
The vehicle’s driving trajectories of three controllers in the experiment: **(A)** Fuzzy back-stepping controller; **(B)** Back-stepping controller; **(C)** Stanley controller.

**Figure 12:**
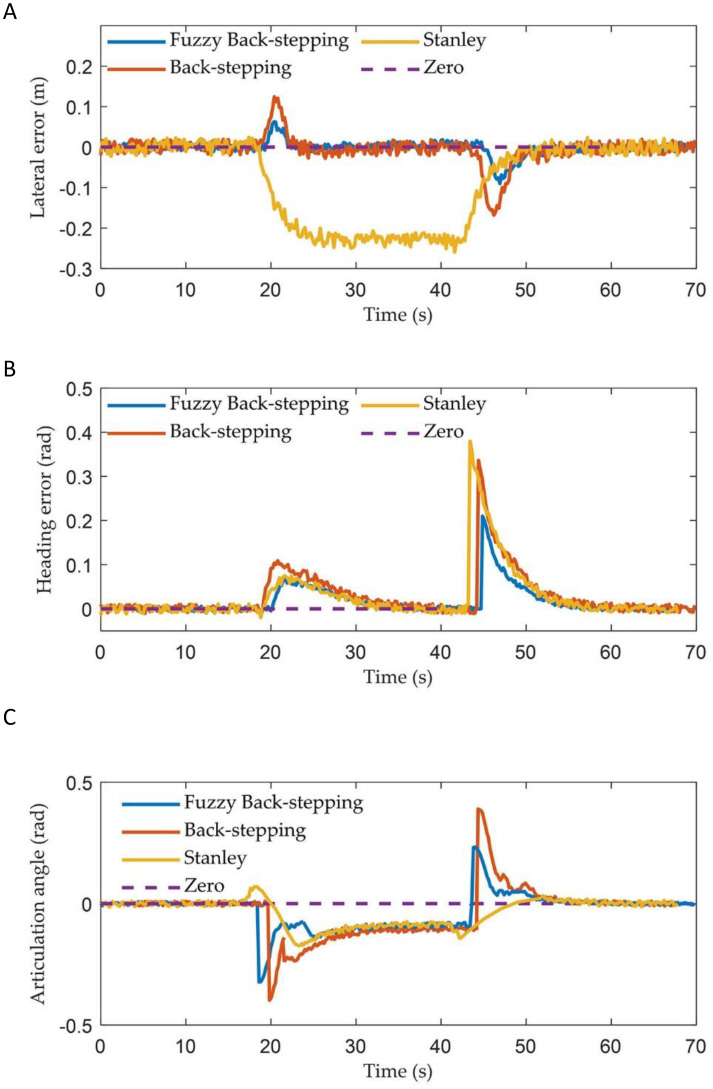
The vehicle states of three controllers in experiment: **(A)** The lateral error; **(B)** The heading error; **(C)** The articulation angle.

**Figures 11** and **12** illustrate the path tracking trajectories and error data of the trailer, demonstrating the performance of the three controllers.

Quantitative statistics of the lateral errors for the three controllers are presented in **Table 3**.

**Table 3:** Statistics of absolute lateral and heading error of three controllers.

Controller	Absolute lateral error/m, and absolute heading error/rad				
	Maximum	MAE	Root-mean-square	Standard deviation	IAE
Fuzzy back-stepping	0.090, 0.175	0.010, 0.016	0.018, 0.031	0.014, 0.027	1.989, 2.916
Back-stepping	0.168, 0.269	0.019, 0.024	0.034, 0.047	0.028, 0.040	3.423, 4.274
Stanley	0.259, 0.380	0.087, 0.030	0.131, 0.066	0.098, 0.060	14.920, 5.162

## 4 Discussion

As illustrated in **Figures 6** and **7**, the two controllers proposed in this article successfully achieved our control objective during the simulation of straight path tracking; specifically, the trailer is able to track the reference path before the tractor engages. In contrast, the traditional Stanley controller permits the trailer’s tracking error to asymptotically converge only after the tractor has come online. In terms of online speed, the back-stepping controller proposed in this paper made the trailer online when the x-coordinate was approximately 12 m, which was comparable to the traditional Stanley controller. However, the proposed fuzzy back-stepping controller, which incorporates a fuzzy adaptive gain coefficient as outlined in Equation 19, exhibited the fastest convergence speed while effectively minimizing oscillations during overshoot. Notably, the trailer had come online at an x-coordinate of approximately 8 m. The proposed controller effectively reduced the trailer’s online time by 36.33% and minimizes overshoot by 68.29%. The MAE and the IAE indicators for lateral and heading errors of the controller proposed in this paper demonstrated superior performance compared to the Stanley controller. This improvement was attributed to our thorough analysis of both the tractor and trailer systems during the model establishment and algorithm design phases, rather than focusing solely on the tractor.

As illustrated in **Figures 8** and **9**, when the ATTV tracks a curved path, a notable phenomenon occurs: the trajectories of the tractor and the trailer do not overlap. This observation aligns with the description provided at the beginning of the introduction. In such scenarios, only one of the two—either the tractor or the trailer—can successfully adhere to the reference path. Our control objective is to ensure that the trailer accurately adheres the reference path. The results of the curve path tracking simulation showed that the controller incorporating the fuzzy adaptive gain coefficient achieved the best tracking performance. This was followed by the original back-stepping controller, which exhibited oscillations after tracking the reference path. In contrast to straight line tracking, the Stanley controller, designed for tractors, failed to reduce the trailer’s lateral error to zero when following a curved path, leading to significant static errors between the trailer’s motion trajectory and the reference path. The MAE and the IAE for both lateral and heading errors of the back-stepping controller proposed in this paper demonstrated significant improvements compared to the Stanley controller. The inclusion of the fuzzy adaptive control gain coefficient had resulted in significant improvements across various metrics of the fuzzy back-stepping controller, which can be attributed to the enhanced dynamic performance of the algorithm. This clearly illustrated the superiority of the controller presented in this paper.

As illustrated in **Figures 11**, **12**, and **Table 3**, the controller proposed in this paper enabled the ATTV-like vehicle to respond quickly and effectively reduces the convergence time of path tracking when transitioning between straight and curved segments. During the initial phase, when the vehicle is stable on the straight path segment, the various errors associated with the three controllers showed minimal differences. Upon reaching the corner of the U-turn path at approximately 20 seconds, the two back-stepping controllers proposed here exhibited lateral errors of about 0.06 m and 0.14 m, respectively, due to the abrupt switching of the reference path index points. However, influenced by the front wheel steering angle output by the algorithm, these errors rapidly converge to within 0.03 m. In contrast, the traditional Stanley controller only accounted for the position of the tractor, thereby allowing it to track the reference curve path. Consequently, the trailer experienced a fixed lateral error of approximately 0.22 m that cannot be mitigated, which aligned with the simulation results. At the juncture where the curve of the U-turn path transitions into a straight line, the two proposed back-stepping controllers quickly converge to within 0.03 m after encountering lateral errors of -0.09 m and -0.18 m, respectively. The traditional Stanley controller, on the other hand, can only achieve gradual convergence of the trailer after the vehicle has completely transitioned into a straight path. Naturally, the performance indicators presented in **Table 3** for the two back-stepping controllers designed for the ATTV outperformed those of the Stanley controller. By incorporating a fuzzy adaptive gain coefficient, the dynamic performance of the controller was enhanced. It demonstrated a rapid convergence in response to large errors while maintaining a minimal overshoot, resulting in the most favorable performance index. In summary, the Stanley controller effectively facilitated asymptotic convergence and path tracking of the tractor, demonstrating its suitability for operations involving tractors equipped with “three-point linkage” implements. However, for ATTVs employing a “single-point hitch” system, the transmission of traction force at the articulation point does not ensure consistency between the travel paths of the trailer and the tractor. This highlights the need for designing a path tracking controller for ATTVs, as discussed in this paper.

## 5 Conclusions

This paper presents a novel fuzzy back-stepping control strategy designed for the path tracking control of ATTVs. Recognizing the phenomenon in which tractors and trailers do not follow the same trajectory during movement, we establish a kinematic error model for the trailer utilizing the velocity decomposition method. To ensure that the trailer adheres to the reference path, we calculate the target front wheel steering angle of the tractor by integrating the back-stepping method with fuzzy logic. The advantage of the designed controller is that the tracking error of the trailer can quickly converge to zero, regardless of whether the path is straight or curved. Results from simulations and semi-physical experiments indicate that the proposed fuzzy back-stepping approach significantly enhances the trailer’s tracking accuracy and speed, particularly on curved paths. This advancement addresses the challenges previously encountered by traditional tractor path tracking methods, where the trailer struggled to follow the reference path. The limitation of our current work is that when the surface of farmland soil becomes excessively slippery, the vehicle may slip, which adversely affects the tracking performance of the algorithm. In our future research, we will concentrate on integrating the fuzzy back-stepping technique with state observation theory, thereby enhancing the algorithm’s generalizability across diverse terrains. Furthermore, we are committed to implementing these technologies in field vehicle trials at the earliest opportunity.

## Data Availability

The original contributions presented in the study are included in the article/supplementary material, further inquiries can be directed to the corresponding author/s.

## Appendix A

To prevent the occurrence of the “jack-knife” phenomenon, it can be derived from geometric relationships that:

(21)
$$\left|\lambda \right|<\frac{\pi -\beta }{2}$$

(22)
$$R_{b}=\frac{L^{h}+L^{f}\text{cos}\lambda }{\text{sin}\lambda }$$

By substituting Equation 21 into 22, it can be derived that:

(23)
$$\left|R_{min}^{b}\right|=\left(L^{h}+L^{b}\text{cos}\frac{\pi -\beta }{2}\right)/\text{sin}\frac{\pi -\beta }{2}$$

where $R_{min}^{b}$ is the minimum turning radius of the planned reference path.

Furthermore, the turning radius of the trailer can also be represented as:

(24)
$$R_{b}=\sqrt{{R_{t}}^{2}+L^{h^{2}}-L^{b^{2}}}$$

where $R_{t}$ is the length from point O to the center of the tractor’s rear axle.

Based on the geometric relationship of tractor steering, $R_{t}$ can be expressed as:

(25)
$$R_{t}=\frac{L^{w}}{2}+\frac{L^{f}}{\text{tan}\delta_{f}}$$

where $L^{w}$ is the tractor front wheelbase.

By substituting Equation 24 into Equation 25, the mathematical relationship between the turning radius of the trailer and the front wheel steering angle of the tractor can be obtained:

(26)
$$R_{b}=\sqrt{{\left(L^{f}\text{cot}\delta_{f}+\frac{L^{w}}{2}\right)}^{2}+L^{h^{2}}-L^{b^{2}}}$$

The maximum front wheel steering angle $\delta_{max}^{b}$ of the tractor can be obtained from Equations 23 and 26 as follows:

(27)
$$\left|\delta_{max}^{b}\right|=\text{arccot}\left\{\frac{1}{L^{f}}\left(\sqrt{{R_{min}}^{2}-L^{h^{2}}+L^{b^{2}}}-\frac{L^{w}}{2}\right)\right\}$$

Furthermore, tractors are limited by their intrinsic mechanical structures, which establish a maximum front wheel steering angle $\delta_{max}^{f}$ and a minimum turning radius $R_{min}^{f}$ upon leaving the factory.

In summary, prior to path tracking, the following physical constraints must be imposed on the curvature of the planned reference path and the steering angle of the tractor’s front wheel:

(28)
$$\text{s.t.}\{\begin{array}{c} \left|R\right|>max\left\{\left|R_{min}^{b}\right|,\left|R_{min}^{f}\right|\right\}\\ \left|\delta_{f}\right|<min\left\{\left|\;\delta_{max}^{b}\right|,\left|\;\delta_{max}^{f}\right|\right\} \end{array}$$

## Appendix B

Substitute Equation 14 into 13:

(29)
$${\dot{V}}_{1}=-\frac{v_{x}^{b}x_{2}}{L^{b}}sat\left(x_{2}\right)\leq 0$$

Based on the Barbalat Lemma (Farkas and Wegner, 2016), it can be inferred that the subsystem ${\left[x_{1},x_{2}\right]}^{T}$ is asymptotically stable.

Substitute Equation 18 into Equation 17:

(30)
$${\dot{V}}_{2}=-\rho_{2}\xi^{2}<0$$

As t→∞ , it follows that $x_{3,r}\left(t\right)=x_{3}\left(t\right)$ . Given that the subsystem ${\left[x_{1},x_{2}\right]}^{T}$ is asymptotically stable, we can assert that $x_{3,r}\left(t\to \infty \right)=0$ . Consequently, it can be concluded that as t→∞ , $x_{3}\left(t\right)=0$ . In summary, when the control input u is given by Equation 26, the system described by Equation 18 is asymptotically stable.
